# Hydrodynamic object identification with artificial neural models

**DOI:** 10.1038/s41598-019-47747-8

**Published:** 2019-08-02

**Authors:** Sreetej Lakkam, B. T. Balamurali, Roland Bouffanais

**Affiliations:** Singapore University of Technology and Design, 8 Somapah Road, Singapore, 487372 Singapore

**Keywords:** Biological physics, Fluid dynamics, Mechanical engineering

## Abstract

The lateral-line system that has evolved in many aquatic animals enables them to navigate murky fluid environments, locate and discriminate obstacles. Here, we present a data-driven model that uses artificial neural networks to process flow data originating from a stationary sensor array located away from an obstacle placed in a potential flow. The ability of neural networks to estimate complex underlying relationships between parameters, in the absence of any explicit mathematical description, is first assessed with two basic potential flow problems: single source/sink identification and doublet detection. Subsequently, we address the inverse problem of identifying an obstacle shape from distant measures of the pressure or velocity field. Using the analytical solution to the forward problem, very large training data sets are generated, allowing us to obtain the synaptic weights by means of a gradient-descent based optimization. The resulting neural network exhibits remarkable effectiveness in predicting unknown obstacle shapes, especially at relatively large distances for which classical linear regression models are completely ineffectual. These results have far-reaching implications for the design and development of artificial passive hydrodynamic sensing technology.

## Introduction

Obstacle detection and identification is instrumental to fish and many amphibians evolving in aquatic environments. This unique capability of detecting the surrounding environment from pressure and velocity sensing is best exemplified by some Mexican blind cave fish that are capable of mapping cluttered areas only by means of hydrodynamic sensing using their lateral-line system (LLS)^1–4^.

For engineered vehicles, the ability to identify obstacles and fluid dynamic conditions of interest can enable efficient path planning while exploiting favorable environment dynamics^5^. Sonar, acoustic Doppler current profiler, and LiDAR are traditionally used to sense and track environmental features (e.g., obstacles, other vehicles, etc.). However, these sensors suffer from blind spots and become inoperative in highly confined, turbid and murky environments. Moreover, stealth technologies can render targets of interest invisible. To overcome these limitations, several recent attempts have been made to design artificial flow sensors by mimicking the basic principles of the LLS^4,6–10^. Thanks to rapid developments in MEMS technology, hydrodynamic sensing could soon become a critical feature of autonomous surface and underwater vehicles performing navigation tasks in the marine environment.

Although significant technological advances have been achieved towards the development of an artificial LLS, a full understanding of the underpinning neural data-processing occurring in aquatic animals equipped with a LLS is still lacking. To date, two basic hydrodynamic models of obstacle identification have been proposed based on two different hierarchical expansions of the flow field^11,12^. Sichert *et al*. introduced a hydrodynamic multipole expansion of the velocity potential and used a maximum-likelihood estimator of linearized relations to estimate location and shape of the obstacle^11^. Bouffanais *et al*. proposed an obstacle representation using a conformal mapping approach combined with a general normalization procedure, revealing the progressive perceptual character of hydrodynamic imaging in the potential flow framework^12^. This hydrodynamic object shape representation was subsequently extended to Stokes flow by Bouffanais & Yue^13^. Given the nonlinear character of the relationship between local flow data and obstacle shape parameter, an unscented Kálmán filter—a robust dynamic probabilistic signal filtering technique for highly nonlinear systems—is used to process the pressure data gathered by a moving sensor. However, with both representations, the classical data processing approaches considered—maximum likelihood estimator and dynamic filtering respectively—exhibit serious limitations in terms of their ability to extract the features characterizing the obstacle, and that, even at relatively small distances away from it. This represents a serious impediment to the actual development of effective artificial LLS. This issue should come as no surprise given that in the natural world, the effectiveness of this unique sensory system critically depends on complex neural data-processing within the central nervous system of the organism.

Over the past five years, advanced machine learning techniques, and deep neural networks in particular, have become the prime choice for most problems categorized as intractable by classical data-mining approaches such as linear regression or decision tree classifiers. Indeed, artificial neural networks (ANN) have repeatedly demonstrated their superior performance on a wide variety of tasks including speech recognition, natural language processing, image classification, etc. It is worth stressing that this breakthrough in artificial intelligence is largely due to the ability of performing unprecedented training of these artificial networks owing to a combination of high processing power and availability of excessively large training data sets. In fluid mechanics, ANN have been applied to the study of turbulent flows, as a data-mining tool to build predictive models associated with direct numerical simulations. Specifically, these predictive models have been used to obtain correction factors in turbulent production terms^14^ or to estimate flow uncertainties^15^. ANN have also been employed to improve and facilitate turbulence modeling^16–18^. Deep neural networks have enabled a novel analysis of turbulent flow fields by banking on the higher dimensional data associated with rotational and intermittent turbulent eddies^19^, thereby revealing that ANN are significantly more accurate than conventional Reynolds-averaged Navier–Stokes models. Very recently, ANN have also been used for solving an engineering problem of obstruction detection in flow pipes^20^.

Here, we develop an advanced data-driven model based on ANN to address the shortcomings of previously used data-processing techniques for the problem of hydrodynamic object recognition, within the potential flow context. We first study and quantify the ability of ANN in localizing and characterizing classical potential flow singularities, such as source/sink and doublet. This allows us to evaluate the influence of the design of the sensor array on the overall effectiveness of the ANN. As a second step, we consider the challenging problem of obstacle identification using the shape representation proposed by Bouffanais *et al*.^12^. Remarkably, the ANN are capable of accurately identifying the shape parameters characterizing the obstacle, including at relative large distances away from the latter. Moreover, our ANN-based approach outperforms classical linear regression models, which are shown to be completely ineffectual for the range of cases considered.

## Methods

### General considerations

The problem of obstacle shape identification is intrinsically ill-posed given the lack of an explicit relationship between the local, static, and finite set of sensed data, on the one hand, and the shape of the obstacle, or the characteristics of the potential flow singularities considered on the other hand. For instance, it is expected that the shape parameters become highly sensitive to minute variations in the sensed data as those are extracted from increasingly large distances away from the obstacle. Machine learning techniques are specifically sought after here since they are known to effectively uncover such unknown relationships between system parameters, even in the presence of high sensitivity to input data as is the case here.

The definition and mathematical formulation of the studied problem follow the ones presented in our previous work^12^, albeit with some notable differences. For instance, here we consider that static velocity or pressure sensing is available, whereas a moving pressure sensor was considered in ref.^12^. For the sake of consistency and clarity, some essential elements of ref.^12^ are repeated here, albeit limited to the necessary level of details.

**Figure 1:**
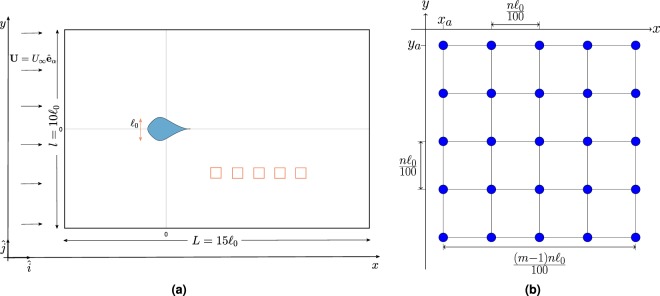
(**a**) Schematic diagram of the computational domain, with the object to be identified shown in blue (characterized by shape coefficients *μ*_1_ = 1/3, *μ*_2_ = 1/6, and *μ*_3_ = 1/12, and the conformal diameter ${\ell }_{0}=2{R}_{0}$). A few examples of possible locations for the sensor array are shown in red. The extracted data is used for training the ANNs. (**b**) Schematic representation of a (*m* = 5, *n*) sensor array with 25 sensing units uniformly separated by $n{\ell }_{0}/100$. The position of the sensor array corresponds to the position (*x*_*a*_, *y*_*a*_) of the top-leftmost sensing unit.

Pressure or velocity sensing are independently available over a static grid-like sensor array located at some adjustable distance away from the obstacle to be identified (see Fig. 1(a)). Note that the mechanosensory LLS is composed of two sensing units (superficial and canal neuromasts) giving animals access to both velocity and pressure sensing^21^. Specifically, the inverse problem consists in estimating as accurately as possible the shape of an obstacle from a *static* sample of flow data. It is worth highlighting that this inverse problem, although closely related to the one in ref.^12^, is considerably more challenging given the small and finite size of the sample of input data considered here to solve this problem. For instance, we show in what follows that a few tens of closely-spaced sensors are amply sufficient to the effectiveness of our data-driven approach. In comparison, sensed flow data are continuously added to the input sample, without any restriction, to achieve accurate hydrodynamic imaging in ref.^12^.

The shape of an obstacle is mathematically related to the sensed data by means of a nonlinear functional that depends on the particular details of the fluid flow around the obstacle. It is worth noting, that the problem is further complexified by the nonlocal relationship between velocity and pressure fields with incompressible flows. Without lack of generality, we consider two-dimensional (2D) potential flows, which is a good representation of three-dimensional shallow-waters with vertical column-like obstacles of unknown shape used by S. Coombs^22^ to study the LLS-based mapping behavior of blind cave fish. Moreover, limiting our analysis to relatively slow fluid flows (maximum speed of the order of one obstacle size ${\ell }_{0}$ per second, see Fig. 1(a)), viscous effects and vortex shedding can be neglected^23^.

### Identification of elementary potential flows

Prior to introducing the complex problem of object shape identification at the core of this work, we first consider the problem of identification of basic singularities, which constitute the building blocks of the potential flow theory, specifically source/sink and doublet. The small number of parameters involved in fully characterizing these singularities make them good candidates for ANN-based recognition. The objective is to use these canonical flows to thoroughly analyze and assess the effectiveness of our data-driven approach (see Results & Discussion) in terms of design of sensor and varying number/nature of parameters to be identified by the ANN.

First, we consider the canonical source/sink flow associated with a velocity potential

1 $$\varphi =\frac{q\sigma }{2\pi }\,\mathrm{ln}\,r=\frac{q\sigma }{2\pi }\,\mathrm{ln}\,\sqrt{{(x-{X}_{s})}^{2}+{(y-{Y}_{s})}^{2}},$$

where *σ* = ±1 for a source (resp. sink), *q* is the strength of the flow (i.e. flowrate per unit depth in 2D), and (*X*_*s*_, *Y*_*s*_) is the location of the singularity in the domain $\bar{{\mathscr{D}}}$ of interest. Given the irrotational character of potential flows, the velocity field is classically given by **v** = *u*(*x*, *y*)**i** + *v*(*x*, *y*)**j** = **∇***ϕ* in 2D. To further simplify the problem, we consider *q* = 2*π*. To fully characterize this potential flow, one has to estimate the triplet $(\sigma ,{X}_{s},{Y}_{s})\in \{\,-\,1,+\,1\}\times \bar{{\mathscr{D}}}$.

The dataset used to train our artificial neural model is obtained by following these steps: (i) generate a large number of samples for the triplet (*σ*, *X*_*s*_, *Y*_*s*_), with random binary values for *σ* and quasi-random low-discrepancy Sobol sequences for both *X*_*s*_ and *Y*_*s*_, (ii) for each sample source/sink (*σ*, *X*_*s*_, *Y*_*s*_) generated, the velocity potential *ϕ* is obtained from Eq. (1), with the *x*-component of the velocity field given by *u*(*x*, *y*) = ∂*ϕ*/∂*x*, and (iii) from the field *u*(*x*, *y*) for each sample (*σ*, *X*_*s*_, *Y*_*s*_), one can calculate the sensed values *u*(*x*_*i*_, *y*_*i*_) at each sensing unit (located at (*x*_*i*_, *y*_*i*_)) of a given array.

The second canonical potential flow considered is the doublet flow, which corresponds to the linear superposition of two closely distant source and sink. In the far-field approximation, the velocity potential is

2 $$\varphi =\frac{\kappa }{2\pi }\frac{\cos (\theta -\alpha )}{r}=\frac{\kappa }{2\pi }\frac{\cos (\theta -\alpha )}{\sqrt{{(x-{X}_{c})}^{2}+{(y-{Y}_{c})}^{2}}},$$

where $\kappa =q\ell$, with $\ell$ being the distance between source and sink, both of strength *q* (see Eq. (1)). The center of the doublet is located at (*X*_*c*_, *Y*_*c*_), and at equal distance of source and sink. The angle *α* is the doublet orientation, measured from the *x*-axis corresponding to *θ* = 0 in domain $\bar{{\mathscr{D}}}$. To further simplify the problem, we consider *κ* = 2*π*. To fully characterize this doublet flow, one has to estimate the triplet $(\alpha ,{X}_{c},{Y}_{c})\in (\,-\,\pi ,\pi ]\times \bar{{\mathscr{D}}}$. It appears therefore clearly that the increased complexity in identifying this doublet flow as compared to a source/sink one, comes from the estimation of *α* as opposed to the simpler binary classification for *σ*.

The process to generate the training dataset for this doublet flow is essentially the same as the one for the source/sink flow, except for the fact that in step (i), one has to generate a large number of samples for the triplet (*α*, *X*_*c*_, *Y*_*c*_), with quasi-random low-discrepancy Sobol sequences for *α*, *X*_*c*_ and *Y*_*c*_. The other steps being identical.

### Object shape representation

We now consider the problem of identifying of the shape of a single obstacle placed in a potential flow. The set of 2D located shapes is infinite dimensional, thereby stressing the inherent challenges associated with such shape classification process^24^. Before proceeding, we note that our work is aimed at classifying shapes while considering the size and location of the obstacle as known quantities. This assumption admits two justifications. First, Burt de Perera^2^ has proved that blind cave fish encode independently in its spatial map obstacle size and location on the one hand, and obstacle shape on the other hand. Second, our previous work^12^ proved that obstacle size *R*_0_ and location *a*_0_ (using the exact same notations as the ones previously introduced) can relatively easily be determined, even at large distances away from the object.

The powerful and compact shape representation introduced in ref.^12^ rests upon a conformal mapping technique of the fluid domain ${\mathscr{D}}$ exterior to the 2D curve limiting the body of the obstacle ${\mathscr{O}}$, in the complex *z*-space with $\bar{{\mathscr{D}}}={\mathscr{D}}\cup {\mathscr{O}}$. Without loss of generality, we assume that the obstacle to be identified is centered—i.e., the conformal center is *a*_0_ = 0—and has a unit size—i.e., the conformal radius is unity *R*_0_ = 1 and typical object size ${\ell }_{0}=2{R}_{0}=2$. As a consequence, the inverse exterior Riemann mapping *z* = *f*^−1^(*ξ*) = *h*(*ξ*) from the exterior of the unit disc |*ξ*| > 1 onto ${\mathscr{D}}$ takes the form of the following Laurent series, with a simple pole at infinity:

3 $$z=h(\xi )=\xi +\frac{{\mu }_{1}}{\xi }+\frac{{\mu }_{2}}{{\xi }^{2}}+\cdots ,\,|\xi | > 1.$$

The key idea behind the use of a Laurent series is that the shape information is encoded into the discrete set of complex coefficients {*μ*_*k*_}_*k*≥1_, also referred to as the shape ‘fingerprint’^24^. Note that the univalent character of the analytic function *h* imposes the following constraints on these coefficients:

4 $$|{\mu }_{k}|\le \frac{1}{\sqrt{k}},\,k\ge 1.$$

As detailed in ref.^12^, each term in 1/*ξ*^*k*^ in Eq. (3) is associated with a specific geometric polygonal perturbation of the unit circle |*ξ*| = 1—specifically a (*k* + 1)-gonal perturbation. For instance, the term in 1/*ξ*^2^ corresponds to an equilateral triangle perturbation, while the one in 1/*ξ*^3^ is of the square type.

Beyond the above mathematical considerations, it is essential to keep in mind that the hierarchical distribution of high-order terms in *h* is directly associated with the progressive perceptual discrimination of the obstacle shape from a distance. Indeed, at large distance from the obstacle, i.e. for |*ξ*| ≫ 1, only a limited number of terms in 1/*ξ*^*k*^ in the asymptotic expansion of *h* are sufficient to provide an accurate approximate shape representation.

We now consider the forward problem associated with any 2D obstacle placed in a uniform external flow ${\bf{U}}={U}_{\infty }{\hat{{\bf{e}}}}_{\alpha }$ making an angle *α* with the *x*-axis (see Fig. 1(a)). The complex potential *w* is obtained through conformal mapping^25^

5 $$w=\varphi +{\rm{i}}\psi ={U}_{\infty }[\xi {{\rm{e}}}^{-{\rm{i}}\alpha }+\frac{{{\rm{e}}}^{{\rm{i}}\alpha }}{\xi }],\,|\xi | > 1,$$

where *ψ* is the streamfunction, and *ξ* = *f*(*z*) is the direct exterior mapping that is uniquely and completely characterized by the shape coefficients {*μ*_*k*_}_*k*≥1_. The expression of *w* in the complex *z*-space is derived using a series inversion of *z* = *h*(*ξ*) (see Eq. (3)), up to a given order. Without loss of generality, we limit our shape fingerprint to cases such that *k* ≤ 3, and our series inversion is expanded up to third order terms in 1/*z*:

6 $$w={U}_{\infty }[z{{\rm{e}}}^{-{\rm{i}}\alpha }+\frac{{\varpi }_{1}{{\rm{e}}}^{{\rm{i}}\alpha }}{z}+\frac{{\varpi }_{2}{{\rm{e}}}^{2{\rm{i}}\alpha }}{{z}^{2}}+\frac{{\varpi }_{3}{{\rm{e}}}^{3{\rm{i}}\alpha }}{{z}^{3}}]+{\mathscr{O}}(\frac{1}{{z}^{4}}),$$

with *ϖ*_1_ = 1−*μ*_1_e^−2i*α*^, *ϖ*_2_ = −*μ*_2_e^−3i*α*^, and ${\varpi }_{3}={\mu }_{1}{{\rm{e}}}^{-2{\rm{i}}\alpha }-({\mu }_{1}^{2}+{\mu }_{3}){{\rm{e}}}^{-4{\rm{i}}\alpha }$. Note that Eq. (6) for *w* ceases to be valid at too short distances away from the considered obstacle. However, this limitation has absolutely no impact on the present study given that both training and classification occur at sufficiently large distances away from the obstacle. The complex flow velocity is classically obtained by

7 $$u+{\rm{i}}v=\frac{{\rm{d}}w}{{\rm{d}}z},$$

giving access to the *x*- and *y*-component of the velocity field **v**. Lastly, the normalized pressure is cast as

8 $$p(z)=-\,\frac{1}{2}{|\frac{{\rm{d}}w}{{\rm{d}}z}|}^{2}.$$

Equations (7) and (8) constitute the analytical solution to the forward problem associated with our inverse problem of shape identification.

Although the process of generating the training dataset for this forward problem is built on similar steps as those detailed in previous sections, it is detailed in the next section due to its technicality.

### Data generation

All lengths are considered in units of the object size ${\ell }_{0}$. For the source/sink/doublet identification cases, the domain is a square one of size $10{\ell }_{0}\times 10{\ell }_{0}$, uniformly discretized with a grid of 1000 × 1000 points. For the object shape identification problem, we consider a rectangular domain of size $15{\ell }_{0}\times 10{\ell }_{0}$, uniformly discretized with a grid of 1500 × 1000 points along the *x*- and *y*-direction respectively. Moreover, all objects are considered to have the same size ${\ell }_{0}$, are centered at the origin, and only differ in their shape fully characterized by the set of first three coefficients {*μ*_1_, *μ*_2_, *μ*_3_} (see Fig. 1(a)). By construction, the object is symmetric with respect to the *x*-axis, but this symmetry in the problem can be removed by changing the incidence *α* of the upstream uniform flow ${\bf{U}}={U}_{\infty }{\hat{{\bf{e}}}}_{\alpha }$.

The dataset used to train and test our artificial neural network model is obtained by generating a large number of samples (*N* = 10^5^ in total, with 90% samples for training and 10% for testing purposes). Each sample corresponds to a unique obstacle shape fully characterized by the triplet (*μ*_1_, *μ*_2_, *μ*_3_). We obtain the training-cum-testing dataset by generating three quasi-random, low-discrepancy Sobol sequences of size *N* for *μ*_1_, *μ*_2_, and *μ*_3_ while accounting for the individual constraint on each *μ*_*k*_ given by Eq. (4). For each sample, the forward problem presented is solved analytically, thereby yielding both components of the velocity field from Eq. (7), and the dynamic pressure from Eq. (8). It is worth stressing that this step—the generation of the training dataset—is often the limiting one when using deep neural networks applied to hydrodynamics problems. Indeed, the effectiveness of such methods critically depends on having access to significantly large datasets, which is often impractical both experimentally and numerically. The last step is trivial and consists in calculating the sensed values *u*(*x*_*i*_, *y*_*i*_), *v*(*x*_*i*_, *y*_*i*_), and *p*(*x*_*i*_, *y*_*i*_) at each sensing unit (located at (*x*_*i*_, *y*_*i*_)) of a given array.

### Data extraction and feature space projection

The estimation of location for data extraction in flow domain is paramount for training the model. Data capturing the whole range of variations in flow field aids in obtaining accurate relations between the flow velocity vector field and the shape coefficients. An important element for data extraction is the design of the sensor, i.e. number of data points and the spacing between the data points. A detailed analysis is performed to determine an adequate sensor array design, comparing prediction model performance in identification of elementary potential flows (see Results & Discussion).

Upon selecting an appropriate array design and its location for data extraction, one can start preprocessing the training-cum-testing dataset. Feature projection methods are known to drastically improve the performance of classifiers susceptible to the Hughes phenomenon^26^. For our problem, this approach is particularly helpful because the intrinsic dimensionality of data is much less than the number of features dealt with. Specifically, we use the classical linear principal component analysis (PCA), which is widely used in modern data analysis^27^. Over the past decade, numerous groups have successfully combined PCA with ANNs to solve a wide range of problems across multiple fields^28–31^. Essentially, PCA maps data to a low-dimensional space while maximizing data variance in the low-dimensional representation. It is important noting that PCA is sensitive to the relative scaling of the original variables which has important implications to this work since the three shape coefficients (*μ*_1_, *μ*_2_, *μ*_3_) vary in different intervals given the constraints (4). In addition, the shape coefficients {*μ*_*k*_}_*k* = 2,3_, which belong to the interval $[0,1/\sqrt{k}]$, are rescaled to be within the unit interval [0, 1] like *μ*_1_.

In practice, PCA is a principal axis transformation technique that minimizes the correlation of variables in a *p*-dimensional space to a *q*-dimensional subspace with a new basis formed by the linearly uncorrelated principal components—i.e., eigenvectors. PCA arranges these principal components in a reducing order of importance in representing input data information. The first component represents the highest information content while the last one contains the least. After obtaining the principal components, the new variables for each sample data are calculated as a linear combination of the original data variables and the higher-order terms can be neglected for dimensional reduction^32^.

The explained variance ratio of a given principal component—ratio between the variance of that component and the total variance—reveals how much information can be attributed to each principal component. This is important as we convert a 9-dimensional input space into a 3-dimensional one. PCA is only used for our central problem of object shape identification, for which the higher dimensional velocity input is mapped onto a lower dimensional space without any other data standardization. By estimating the explained variance ratio, we can see that the highest explained variance for the first principal component is approximately 95.1%, while it is 4.88% for the second principal component contains, and the third principal component has a very low explained variance of 0.02%. Together, the three principal components represent almost 100% of the information, while the first two, with 99.98%, constitute the bulk of it. These results are obtained with a square sensor array comprised of 25 sensing points (optimal (5, 10) design identified at a data extraction distance $2.8{\ell }_{0}$ behind the object (leftmost red square in Fig. 1(a)).

The crucial need for preprocessing the dataset by means of PCA is apparent when plotting the estimated values of the shape coefficients ${\{{\tilde{\mu }}_{k}\}}_{k=1,2,3}$ against the actual ones {*μ*_*k*_}_*k* = 1,2,3_ with and without PCA (see Supplementary Material, Note I). It is worth pointing out that for the identification of elementary potential flows (source/sink and hydrodynamic doublet) no PCA or any data standardization technique have been used.

### Prediction model

As already mentioned, artificial neural networks are chosen here given their vastly superior performance in dealing with implicit nonlinear relationships as compared to traditional methods^33^. For instance, with linear regression (LR), nonlinearities have to be known a priori and expressed explicitly. ANN, however, do not require any a priori knowledge of possible relations between parameters, and typically infer these relations much more effectively than multiple regression analyses^34^, or other conventional statistical methods^35^. Hence, ANN provide a nonparametric approach that is free of assumptions and adaptive, i.e., if new data is available, the model will adapt to it.^36^.

Whenever ANN are used, one should always verify that traditional data-processing methods such as LR are indeed ineffectual. We therefore evaluate the effectiveness of a classical LR approach in dealing with the simple elementary doublet flow (see Methods). To further simplify the problem, we fix the orientation of the doublet to *α* = 0 and the goal is to estimate the location (*X*_*c*_, *Y*_*c*_) of the center of the doublet. Figure S3 (see Supplementary Material, Note III) clearly shows that linear regression is absolutely ill-equipped for this basic task. In what follows, the effectiveness of all prediction models is quantified by means of the relative error between estimated value $\tilde{{\rm{\Sigma }}}$ and the actual real value Σ of any given property of the system considered: Σ = *X*_*s*_, *Y*_*s*_, *σ*, *X*_*c*_, *Y*_*c*_, *α*, *μ*_*k*_, etc. This relative error, denoted *ρ*, is defined as

9 $$\rho =\frac{\Vert \tilde{{\rm{\Sigma }}}-{\rm{\Sigma }}\Vert }{\Vert {\rm{\Sigma }}\Vert },$$

and has values in the unit interval.

Given the complete ineffectiveness of LR, artificial neural networks are used to estimate the object shape coefficients. The ANN architecture considered here contains three hidden layers with *m* = 100, 100, and 16 hidden units ${\{{H}_{i}^{l}\}}_{i=1,\ldots ,m}$ per hidden layer *l* respectively (see Fig. S2, Supplementary Note II). In addition, a rectified linear unit (ReLU) serves as activation function. In the absence of such an activation function, the neural networks exhibit poor performance in most of the cases considered, including with the basic source/sink identification problem (not shown here). The ReLU activation approach is known to be more effective than other classical continuous activation functions, e.g. sigmoid or hyperbolic tangent^37,38^.

The so-called ‘Adam’ method, which is an extension of the classical gradient-descent algorithm, is used to stochastically optimize the weights of the neurons as part of the backpropagation algorithm^39^. Backpropagation acts iteratively following a two-step sequence consisting of a forward pass that is followed by a backward one. In the former step, activation value for each neural unit in the network are calculated from the weights of the adjacently connected neurons. During the backward pass, weights are corrected based on the difference between generated output during the forward pass and the desired output.

The ANN architecture described above is implemented by means of the scikit-learn machine learning library^40^. An ANN with a single unit in the output layer, combined with a sigmoid activation and a logarithmic loss function is used for the binary classification of *σ* = ±1 for the source/sink identification problem (see Eq. (1)). An ANN regressor model with two units in the output layer is used to estimate the location of the flow singularity (*X*_*s*_, *Y*_*s*_) (resp. (*X*_*c*_, *Y*_*c*_)) for the source/sink (resp. center of the doublet). An additional ANN regressor with one unit in the output layer is used to estimate the orientation *α* of the doublet (see Eq. (2)). For the object shape identification problem, an ANN with three units in the output layer is used for the regression task of estimating the three shape coefficients {*μ*_*k*_}_*k* = 1,2,3_, with each unit corresponding to a given shape coefficient (see Supplementary Material, Fig. S2). All the ANN models used for regression tasks are based on a linear activation in the output layer with a squared-error loss function for optimization. As already mentioned, for each case reported in this study, the total number of samples is *N* = 10^5^, which are generated through the process detailed previously. As the samples are generated using a quasi-random sequence, the dataset is randomly split with 90,000 samples forming the training set, and the remaining 10,000 samples are used for testing. All ANN models are trained with a learning rate of 0.001, which has been found to yield a consistent reduction in error-loss between epochs. Further, the model uses a mini-batch size of 200 for 200 epochs. No early stopping was used to avoid having a different total number of epochs for various model trainings, which would have made our results harder to reproduce.

## Results and Discussion

### Influence of the sensor array design

Unsurprisingly, among the animal taxa afforded with a mechanosensory apparatus enabling flow detection, there exists a wide range of natural designs: from the LLS in fish, to patches of whiskers in pinipeds, etc. One is therefore led to question the influence of the design of the sensor array on the effectiveness of the hydrodynamic object identification at a distance. It is worth pointing out that this important question has relevance at the practical MEMS-device design level, but also at the fundamental data-processing level, which is the scope of this work.

We consider square sensor arrays fully characterized by a set of integers (*m*, *n*), having *m*×*m* sensing units evenly separated by a spacing of length $n{\ell }_{0}/100$. The side length of these square arrays is simply $(m-1)n{\ell }_{0}/100$, expressed in units ${\ell }_{0}$ of the object size (see Fig. 1(b)). For instance, the (5, 10) array comprises of 25 sensing units evenly separated by ${\ell }_{0}/10$. The position (*x*_*a*_, *y*_*a*_) of the sensor array is measured from the origin (conformal center of the object as in Fig. 1(a)) and corresponds to the position of the top-leftmost sensing unit. By varying *m* and *n*, we can generate a wide range of sensor arrays of different sizes/resolutions.

**Figure 2:**
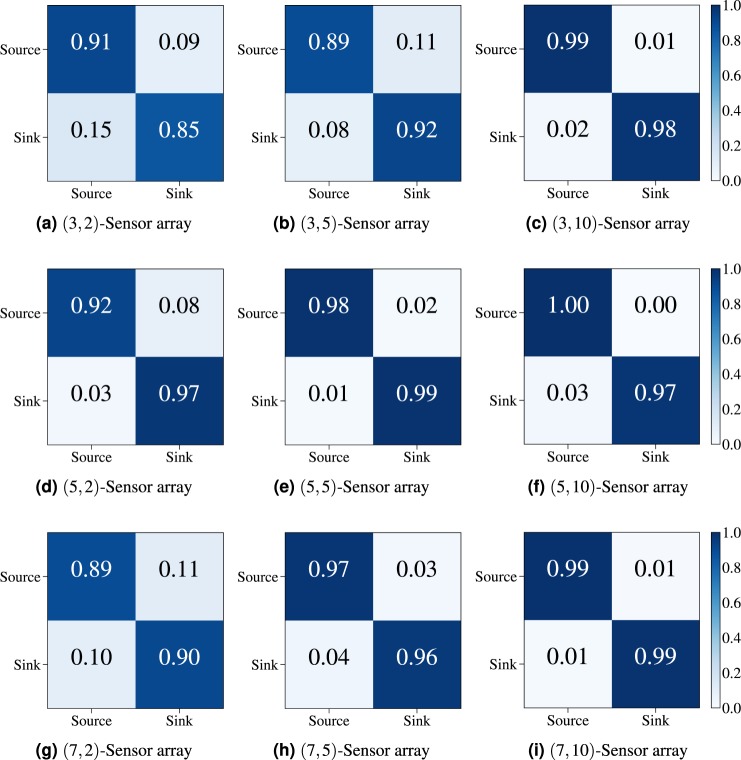
Confusion matrices for 9 distinct (*m*, *n*)-sensor arrays performing an ANN-based binary classification between source/sink flows. Actual flow singularity is on the *x*-axis, while estimated singularity is on the *y*-axis. The numerical entries are percentages also shown using a blue color map of the unit interval.

We first tackle the ANN-based source/sink flow identification problem (see Methods) with 9 distinct sensor arrays. It is worth recalling that for this problem the ANNs are trained with the *x*-component of the velocity field, *u*, extracted at the *m*^2^ sensing points, with the array located at $({x}_{a}=2{\ell }_{0},{y}_{a}=-\,2{\ell }_{0})$ measured from the origin located at the center of the square domain of side length $10{\ell }_{0}$. To assess the performance of the binary classifier distinguishing between source (*σ* = +1) and sink (*σ* = −1), we present the results using confusion matrices^33^. These are 2-by-2 tables, that report the fraction of false positives, false negatives, true positives, and true negatives, thereby providing an in-depth report of the statistical classification beyond just the proportion of correct estimations. Essentially, an effective binary classification leads to a mostly diagonal confusion matrix (i.e. with high fractions of true positives and true negatives). Off-diagonal entries represent the percentage of misclassified samples (i.e. false positives and false negatives). Figure 2 shows the confusion matrices corresponding to 9 sensor arrays obtained with *m*^2^ = 9, 25, 49 sensing units and *n* = 2, 5, 10. For instance, the (3, 2)-sensor array (see Fig. 2(a)) yields on average a 91% accuracy in identifying sources, 85% for sinks, 15% misclassification of sinks, and 9% misclassification of sources. Although we notice a systematic improvement when going from a 3 × 3 array to a 5 × 5 one, further doubling the number of sensing units (7 × 7) leads to a slight reduction in the effectiveness of the classification, and that for all three values of *n*. This effect is attributed to the fact that this binary classification is carried out using data sets obtained from the continuous field *u*(*x*, *u*). Classification tasks using discrete data sets are usually more effective. As expected, increasing *n* almost always yields a higher accuracy, which can easily be explained by the increased size of the sensor array.

**Figure 3:**
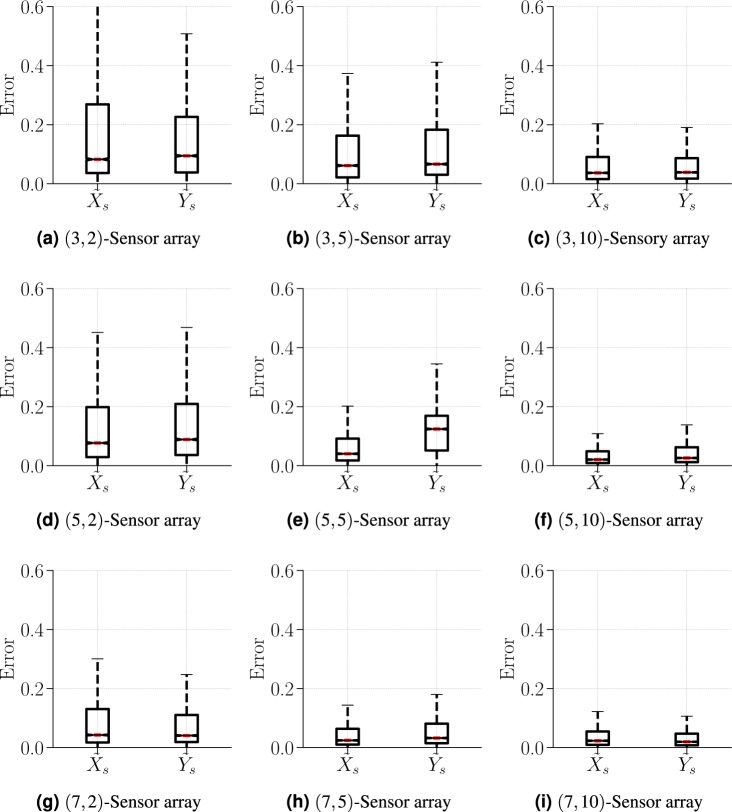
Performance of the ANN-based regressor in estimating the location of the flow singularity (*X*_*s*_, *Y*_*s*_). The distribution of the relative error is shown by means of boxplots representing the the median error in red, and box extremities corresponding to the 25^th^ and 75^th^ percentiles of the distribution of error, and whiskers being at the 10^th^ and 90^th^ percentiles.

Next, we turn to the effectiveness of the regressor in finding the location (*X*_*s*_, *Y*_*s*_) of the flow singularity (source or sink). Figure 3 shows the relative error (see Eq. (9)) in the estimated position ${\tilde{X}}_{s}$ and ${\tilde{Y}}_{s}$, with boxplots representing the the median error in red, and box extremities corresponding to the 25^th^ and 75^th^ percentiles of the distribution of error, and whiskers being at the 10^th^ and 90^th^ percentiles. Contrary to the previous binary classification, the ANN-based regressor exhibits a systematic improvement with increases in both *m* and *n*. This is particularly clear when observing the steep narrowing of the whiskers of the distribution of relative error. An increase in *n*, however, yields a more pronounced improvement of the overall accuracy of the regressor. This can easily be attributed to the fact that the associated array design reduces data localization. The difference in performance between classifier and regressor can be traced to the continuous nature of the field *u*(*x*, *y*). Again, classifiers are known to be more effective when learning from discrete data sets.

From Figs 2 and 3, we can conclude that the sensor array (*m* = 5, *n* = 10) (with 25 sensing units and side length $0.4{\ell }_{0}$) constitutes a satisfactory option given its high performance as classifier, as well as regressor. For instance, the array (*m* = 7, *n* = 10) yields marginally better performance although it comprises of almost twice as many sensing units, and has a sensing area more than doubled that of (*m* = 5, *n* = 10). In all results that follow, the (*m* = 5, *n* = 10)-sensor array design is systematically used, unless mentioned otherwise.

**Figure 4:**
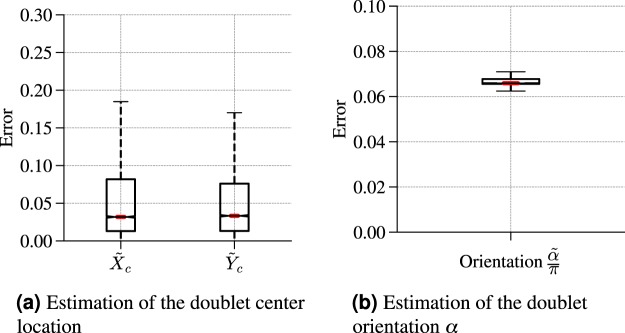
Performance of the ANN-based regressor in estimating the triplet (*α*, *X*_*c*_, *Y*_*c*_) characterizing doublet flows using a (5, 10)-Sensor array. The distribution of the relative error is shown by means of boxplots representing the the median error in red, and box extremities corresponding to the 25^th^ and 75^th^ percentiles of the distribution of error, and whiskers being at the 10^th^ and 90^th^ percentiles.

Next, we use an ANN-based regressor to estimate the triplet (*α*, *X*_*c*_, *Y*_*c*_) characterizing the orientation and center of a doublet flow (see Methods). The distribution of relative error is shown in Fig. 4 when using the flow speed $\sqrt{u{(x,y)}^{2}+v{(x,y)}^{2}}$ to generate the sensed data with the (5, 10)-sensor array located at a $({x}_{a}=2{\ell }_{0},{y}_{a}=-\,2{\ell }_{0})$ from the origin. This 3-parameter regression task being more demanding than the identification of a single source/sink, we observe that the estimation of the doublet center location (*X*_*c*_, *Y*_*c*_) is slightly less accurate than in the single source/sink localization case, with a broader distribution of errors (see Fig. 4(a)). It is also worth noticing that the estimation of the doublet orientation *α* is noticeably more challenging than its localization (see Fig. 4(b)).

### Object shape identification

As a final step, we consider the object shape identification problem, which requires estimating the triplet (*μ*_1_, *μ*_2_, *μ*_3_) of shape coefficients from distant hydrodynamic measurements (see Methods). An important question is which hydrodynamic sensed data yields the best estimation of the triplet. As already mentioned, in the natural world, the LLS constitutes an array of dual mechanosensors (canal and superficial neuromasts) thereby giving access to both pressure and velocity fluctuations. We therefore consider the effectiveness of the ANN-based regression with four different sensed data: (i) the *x*-component *u* of the velocity field, (ii) the *y*-component *v*, (iii) the flow speed $\sqrt{{u}^{2}+{v}^{2}}$, and (iv) (*u*^2^ + *v*^2^), which essentially amounts to the dynamic pressure. The median estimation errors for all four cases are reported in Table 1.

**Table 1:** Median relative error in estimating the shape coefficients (*μ*_1_, *μ*_2_, *μ*_3_) using different sensed data.

Sensed Data	*μ*_1_	*μ*_2_	*μ*_3_
*u*	0.0036	0.0082	0.0181
*v*	0.0051	0.0172	0.0219
$\sqrt{{u}^{2}+{v}^{2}}$	0.0056	0.0127	0.0178
*u*^2^ + *v*^2^	0.0040	0.0077	0.0125

The first column specifies the sensed data serving as model training data. The other three columns report the median relative error in estimating *μ*_1_, *μ*_2_, and *μ*_3_. The results are obtained using a (5, 10)-sensor array located at $({x}_{a}=2{\ell }_{0},{y}_{a}=-\,2{\ell }_{0})$ measured from the origin.

**Figure 5:**
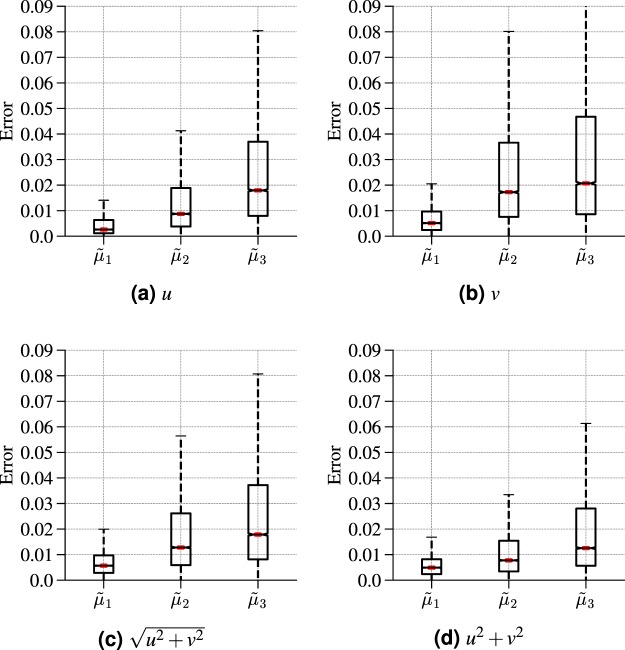
Distribution of relative error in estimating the shape coefficients for all four options of sensed data. The results are obtained using a (5, 10)-sensor array located at $({x}_{a}=2{\ell }_{0},{y}_{a}=-\,2{\ell }_{0})$ measured from the origin.

Interestingly, when considering the dynamic pressure (*u*^2^ + *v*^2^) as the sensed data, the ANN-based regression yields a significant improvement over all other 3 options, and that for all 3 shape coefficients. At this stage, it is worth highlighting that for the sink/source identification case, *u* has been found to be the best option, while for the doublet flow identification, the flow speed $\sqrt{{u}^{2}+{v}^{2}}$ led to the best results. These results are further confirmed in Fig. 5, which shows the full distribution of relative errors for all 4 options of sensed data. We are therefore led to conclude that there is no absolute—i.e. problem independent—best choice for the sensed data, which is consistent with the dual mechanosensory nature of the LLS.

The other important element gathered from Table 1 and Fig. 5 is the hierarchy in error when going from *μ*_1_, to *μ*_2_, and ultimately to *μ*_3_. This observation is consistent with what has previously been reported by Bouffanais *et al*.^12^, and highlights the inherently progressive perceptual shape discrimination capability of hydrodynamic object identification.

**Figure 6:**
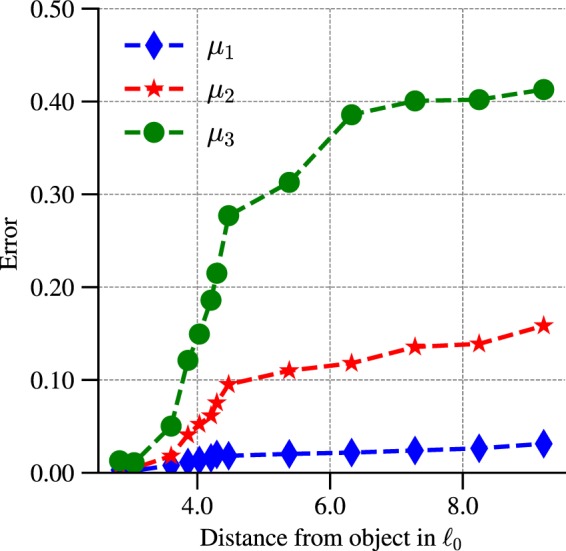
Median relative error in estimating all three shape coefficients when increasing the distance *d* (measured in ${\ell }_{0}$ units) between the sensor array and the object.

**Figure 7:**
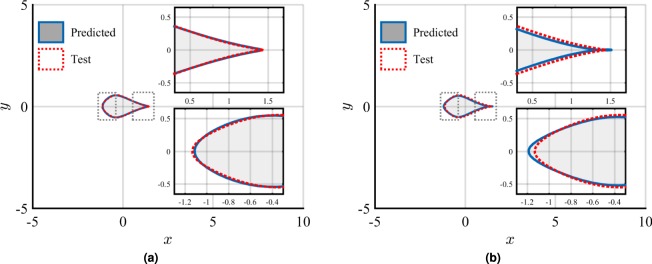
Direct visual comparison of the actual shape of the obstacle (test shape limited by the dotted line corresponding to *μ*_1_ = 1/3, *μ*_2_ = 1/6, and *μ*_3_ = 1/12) with the predicted shape (solid line) at two different distances: (**a**) sensor array in the near-field region for a distance $d=2.8{\ell }_{0}$ (${\tilde{\mu }}_{1}=0.3363$, ${\tilde{\mu }}_{2}=0.1737$, ${\tilde{\mu }}_{3}=0.0711$), and (**b**) sensor array in the far-field region for a distance $d=9.2{\ell }_{0}$ (${\tilde{\mu }}_{1}=0.3517$, ${\tilde{\mu }}_{2}=0.1902$, ${\tilde{\mu }}_{3}=0.1227$).

The final critical factor to be investigated is the influence of the distance between the sensor array and the object whose shape is sought. Previous attempts at detecting and identifying an obstacle by means of hydrodynamic imaging were based on classical data processing approaches exhibiting severe limitations in terms of their ability to extract shape features, even at short distances away from the obstacle^11,12^. Figure 6 shows the performance of the ANN-based regression for all three shape coefficients at various positions behind the obstacle (see some positions of the sensor array highlighted in red in Fig. 1(a)). These results were obtained by sensing the dynamic pressure (*u*^2^ + *v*^2^) with a (5, 10)-sensor array located along the line ${y}_{a}=-\,2{\ell }_{0}$ with *x*_*a*_ varying from $2{\ell }_{0}$ to approximately $10{\ell }_{0}$. On the horizontal axis of Fig. 6, one finds the distance $d=\sqrt{{x}_{a}^{2}+{y}_{a}^{2}}$ between the top-left corner of the array and the conformal center of the obstacle. As expected, the relative error in estimating the shape coefficients {*μ*_*k*_}_*k* = 1,2,3_ increases with the distance *d*. However, the performance of the ANN-based regression is outstanding for *μ*_1_ with less than 5% relative error at very large distances away from the obstacle $(\,\sim \,10{\ell }_{0})$. For the triangular coefficient *μ*_2_, the performance is remarkably good (relative error around 10% at $5{\ell }_{0}$, and slightly above 15% at $9.2{\ell }_{0}$) compared to previously obtained results^11,12^. As anticipated, the estimation of the quadrangular coefficient *μ*_3_ is much more challenging, and this quantity is only reasonably estimated for distances between the sensor array and the obstacle below $4{\ell }_{0}$. However, it is worth doing a direct visual comparison of the actual shape of the obstacle (test shape) with the predicted shape, at two different distances, and that combines the use of all three shape coefficients: (a) sensor array in the near-field region for a distance $d=2.8{\ell }_{0}$, and (b) sensor array in the far-field region for a distance $d=9.2{\ell }_{0}$. The obstacle shapes for both of these cases are shown in Fig. 7. When sensing in the near-field region (Fig. 7(a)), the difference between test shape and predicted one is barely noticeable. Interestingly, when sensing in the far-field region (Fig. 7(b)), although the median relative error in *μ*_3_ is close to 40%, the predicted shape is visually extremely similar to the test shape. For most practical purposes, it may be concluded that the predicted shape is sufficiently close to the test one.

## Conclusion

In this work, we considered the problem of hydrodynamic object identification from remotely sensed flow data in the potential flow framework. We proposed and implemented a neural data-processing model exhibiting vastly superior performance compared to previously considered techniques (maximum likelihood estimator and dynamic filtering). This approach uses artificial neural networks that are trained with a large data set conveniently obtained from the analytical solution to the forward problem associated with our inverse problem of object shape identification.

The effectiveness of our neural networks regression and classification is assessed on two singular potential flows: source/sink flow, and doublet flow. Classical linear regression techniques are found to be completely ineffective in identifying both singularities. The influence of the sensor array design is analyzed, thereby revealing that a relatively small and static array with 25 sensing units is sufficient for the considered task. The ANN-based obstacle identification further confirms the progressive perceptual character of this hydrodynamic shape discrimination capability. It also shows remarkable performance even at relatively large distances away from the obstacle.

Moreover, the ANN-based data-processing methodology reported here is being further developed to tackle complex fluid flow problems involving unstable swirling flows^41^, as well as the identification of complex relationships between flow variables in turbulent channel flows.

Finally, it is important stressing that the combination of our ANN-based data-processing technique with recent hardware advances in MEMS paves the way to the design and development of full-fledged artificial lateral-line systems that could be integrated to the next generation of engineered underwater vehicles and robots.

## Supplementary information

Supplementary Material
