# Supplementary material for: Hydrodynamic object identification with artificial neural models
Source: Sci Rep. 2019 Aug 2;9:11242. doi: 10.1038/s41598-019-47747-8 (PMC6677828; doi:10.1038/s41598-019-47747-8)
Supplement: Supplementary file 1 — Supplementary Material [file 41598_2019_47747_MOESM1_ESM.pdf]

Hydrodynamic object identification with artificial neural models

Sreetej Lakkam, Balamurali B T, and Roland Bouffanais*
Singapore University of Technology and Design, 8 Somapah Road, Singapore 487372

Supplementary Material

I. PRINCIPAL COMPONENT ANALYSIS

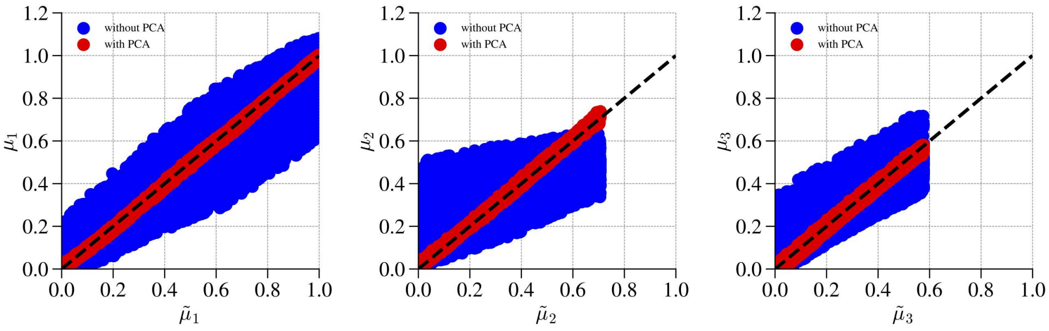

(a) μ_1 distribution (b) μ_2 distribution (c) μ_3 distribution

Fig. S 1. Effect of PCA-based dimension reduction on the prediction of the obstacle shape coefficients. The cloud of points show the estimated values $\{\tilde{\mu}_k\}_{k=1,2,3}$ against the actual ones $\{\mu_k\}_{k=1,2,3}$, while accounting for the hard mathematical constraint $0 < \mu_k < 1/\sqrt{k}$. In all three subfigures, the cloud of blue (resp. red) points are obtained without (resp. with) PCA.

II. NEURAL NETWORK ARCHITECTURE

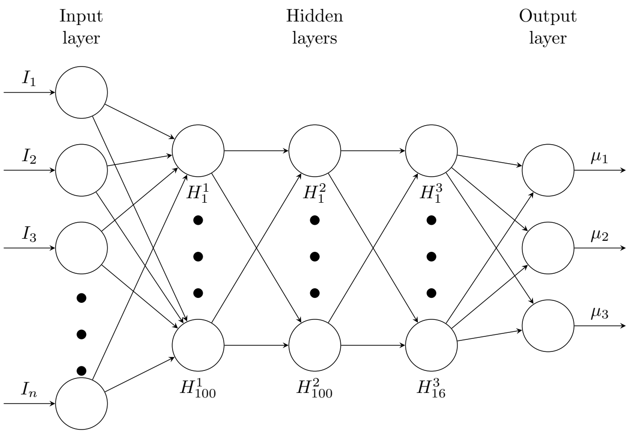

Fig. S 2. ANN architecture used in all cases considered: a fully connected neural network model. At the input layer, we have $\{I_i\}_{i=1,\ldots,n}$ representing the model input data obtained following the data generation process described in the Methods section for the selected sensor array. At the output layer, we have the estimated quantities of interest $\Sigma = (X_s, Y_s, \sigma)$ or $\Sigma = (X_c, Y_c, \alpha)$ for the source/sink or doublet flows respectively, and $\Sigma = (\mu_1, \mu_2, \mu_3)$ for the object shape identification problem. This architecture contains three hidden layers with $m = 100$, 100, and 16 hidden units $\{H_i^l\}_{i=1,\ldots,m}$ per hidden layer l respectively.

* bouffanais@sutd.edu.sg

III. LINEAR REGRESSION VS. ARTIFICIAL NEURAL NETWORKS

It is not uncommon to find studies employing ANN-based regression techniques for problems previously solved with classical linear regression approaches. However, one could ponder the question of what justifies the use of such powerful techniques as ANNs to deal with problems that are tractable by means of LR. Figure 3 shows that for both elementary potential flow problems of source/sink and doublet identifications (see Methods), LR is completely ineffectual (relative error close to 100%) while our ANN-based regression approach yields accurate localization of both singularities (median relative error below 3%).

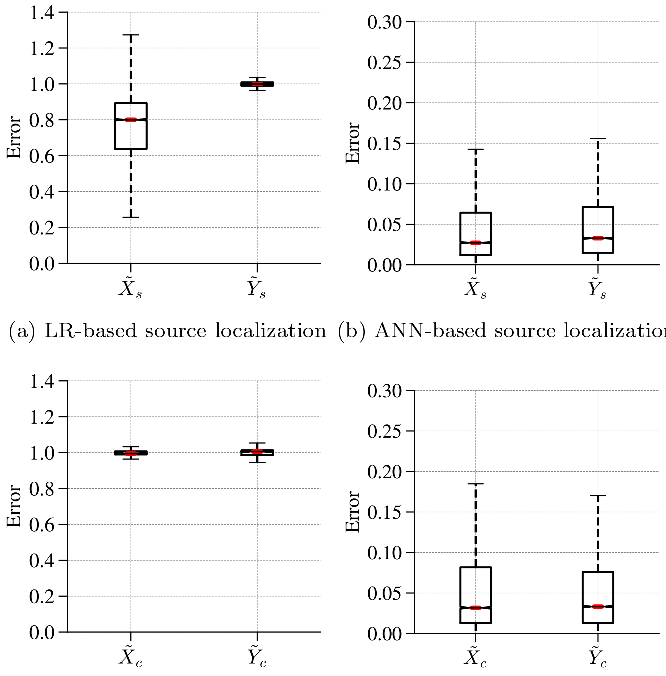

(a) LR-based source localization (b) ANN-based source localization

(c) LR-based doublet localization (d) ANN-based doublet localization

Fig. S 3. Performance comparison of LR-based vs. ANN-based regression in estimating the location of flow singularities (see Methods). The distribution of the relative error is shown by means of boxplots representing the the median error in red, and box extremities corresponding to the 25th and 75th percentiles of the distribution of error, and whiskers being at the 10th and 90th percentiles. The results are obtained using a $(5, 10)$-sensor array located at $(x_a = 2\ell_0, y_a = -2\ell_0)$ measured from the origin.
